# Relaxation Optimized Heteronuclear Experiments for Extending the Size Limit of RNA Nuclear Magnetic Resonance

**DOI:** 10.1021/jacs.4c17823

**Published:** 2025-03-18

**Authors:** Aarsh Shah, Heer Patel, Arjun Kanjarpane, Michael F. Summers, Jan Marchant

**Affiliations:** † Department of Chemistry and Biochemistry, University of Maryland Baltimore County (UMBC), Baltimore, Maryland 21250, United States; ‡ Howard Hughes Medical Institute, University of Maryland Baltimore County (UMBC), Baltimore, Maryland 21250, United States

## Abstract

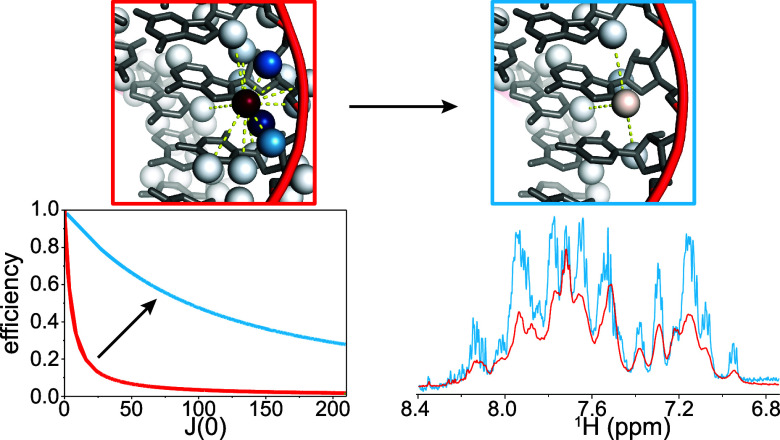

The application of NMR to large RNAs has been limited by the inability to perform heteronuclear correlation experiments essential for resolving overlapping ^1^H NMR signals, determining interproton distance restraints and interhelical orientations for structure calculations, and evaluating conformational dynamics. Approaches exploiting ^1^H–^13^C correlations that are routinely applied to proteins and small RNAs of ∼60 nucleotides or fewer are impractical for larger RNAs due to rapid dipolar relaxation of protons by their attached carbons. Here we report a ^2^H-enhanced, ^1^H–^15^N correlation approach that enables atom-specific NMR characterization of much larger RNAs. Purine H8 transverse relaxation rates are reduced ∼20-fold with ribose perdeuteration, enabling efficient magnetization transfer via two-bond ^1^H–^15^N couplings. We focus on H8–N9 correlation spectra which benefit from favorable N9 chemical shift anisotropy. Chemical shift assignment is enabled by retention of protons at the C1′ position, which allow measurement of two-bond H1′–N9 and through-space H1′–H8 correlations with only a minor effect on H8 relaxation. The approach is demonstrated for the 232 nucleotide HIV-1 Rev response element, where chemical shift assignments, ^15^N-edited nuclear Overhauser effects, and ^1^H–^15^N residual dipolar couplings are readily obtained from sensitive, high-resolution spectra. Heteronuclear correlated NMR methods that have been essential for the study of proteins can now be extended to RNAs of at least 78 kDa.

## Introduction

RNA molecules play essential roles in a variety of biological processes ranging from transcriptional and translational regulation, intracellular trafficking, and enzyme catalysis,^1−4^ in addition to serving as carriers of genetic information.^5−9^ Like proteins, RNAs express their diverse functions via intrinsic structural and dynamical properties dictated by their primary nucleotide sequences. The availability of a vast protein structure database (more than 200,000 three-dimensional protein structure depositions in the Protein Databank)^10^ has facilitated the development of approaches for accurately predicting protein structures and intermolecular interactions.^11−14^ In comparison, RNA structures account for fewer than 1% of all depositions in the PDB. Additional RNA structural information is needed not only for answering mechanistic questions about the large and growing number of biologically important RNAs, but also for development of improved tools for RNA structure prediction and validation. The paucity of RNA structural information can be at least partly attributed to difficulties in applying commonly employed methods for structural characterization. Conformational heterogeneity, flexibility, and a relatively uniform overall negative charge can hinder crystallization and complicate analyses by electron microscopy, and a combination of limited chemical shift dispersion and severe line broadening caused by rapid NMR signal relaxation creates challenges for NMR analyses of large RNAs. For these reasons, while nearly 30% of all RNA structures deposited in the PDB were determined by NMR, most (>95%) of these are for RNAs comprising fewer than 60 nucleotides.

Incorporation of stable isotopes is an essential tool for the study of macromolecules by NMR, allowing for dispersion of chemical shifts into multiple spectral dimensions and serving as probes for structure and dynamics. In RNA, enrichment of ^13^C and ^15^N has allowed the development of a library of experiments for structural and dynamics studies,^15,16^ including for detection of hydrogen bonds,^17−21^ determining torsion angles,^22,23^ measuring relaxation rates,^24−27^ and residual dipolar couplings (RDCs).^28−31^ These approaches can be routinely applied to RNAs up to ∼60 nucleotides but in larger RNAs the loss of signal due to rapid transverse relaxation renders many experiments effectively impossible. In particular, experiments relying on correlation of ^1^H resonances with those of their attached ^13^C nuclei (the predominant heteronuclear correlation in nucleic acids) suffer due to the effect of large dipole–dipole coupling (DD) between these nuclei. The relatively large chemical shift anisotropy (CSA) of ^13^C nuclei allows for mutual DD/CSA cancelation (TROSY),^32^ but this cancellation is not effective for aromatic ^1^H nuclei,^33^ and an upper limit for the effective rotational correlation time of ∼30 ns has been suggested for proton-detected base ^13^C–^1^H TROSY experiments.^28^ Carbon-detected experiments should be applicable to larger RNAs due to the slowly relaxing ^13^C TROSY component.^17,34−36^ This TROSY effect can be improved further by substitution of the attached ^1^H with ^19^F,^37^ in some cases to a remarkable degree,^38^ albeit with CSA-dominant ^19^F relaxation significantly faster than for equivalent ^1^H nuclei.

Uniform incorporation of ^15^N allows for heteronuclear correlation experiments of imino groups via TROSY experiments^39^ which can provide slowly relaxing resonances, although signal broadening due to exchange with the solvent can be substantial.^40^ Experiments using long-range ^1^H–^15^N couplings have also been previously described.^41^ While these couplings are small, the large internuclear distance and low ^15^N gyromagnetic ratio means that ^1^H relaxation is similar for ^15^N-labeled and unlabeled molecules. We previously exploited two-bond couplings from the intrinsically sharp adenosine H2 protons to measure RDCs in large RNAs,^42^ but the limitation to adenosines combined with overlapping ^15^N signals means this approach affords relatively sparse coverage. To address this problem, we have developed an approach in which ^15^N-labeled nucleobases are combined with deuterium-enriched riboses, allowing acquisition of ^1^H–^15^N correlation spectra with high sensitivity and excellent resolution and thus expanding RDC coverage to potentially all G·C and A·U base pairs. We present a theoretical framework for designing appropriate labeling schemes and demonstrate applicability to large systems with the 232 nucleotide RRE from HIV-1 (RRE232).

## Results and Discussion

In the slow motion limit, transverse relaxation of a spin *I* due to dipolar coupling is, to a good approximation
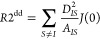


Where *J*(0) is the zero frequency spectral density, the summation runs over all intramolecular spins *S*, where *A*_*IS*_ = 5 for homonuclear and *A*_*IS*_ = 4 for heteronuclear cases, and the dipolar coupling between spins *I* and *S* is given by *D*_*IS*_ = μ_0_γ_*I*_γ_*S*_*ℏ*/4π*r*^3^, with μ_0_ the vacuum magnetic permeability, γ_*x*_ the gyromagnetic ratio of spin *x*, *ℏ* the reduced Planck’s constant, and *r* the internuclear distance. For isotropic rotational diffusion *J*(0) = τ_*c*_, the rotational correlation time. For a given structure and isotope composition, the remaining terms can be explicitly calculated for each spin *I* of interest and will give *R*2/τ_*c*_; i.e. the contribution to transverse relaxation in Hz/ns of rotational correlation time, giving an easily interpretable description of the effect of different isotope compositions on how sensitive transverse relaxation of a given structure is to increasing molecular weight.

**Figure 1:**
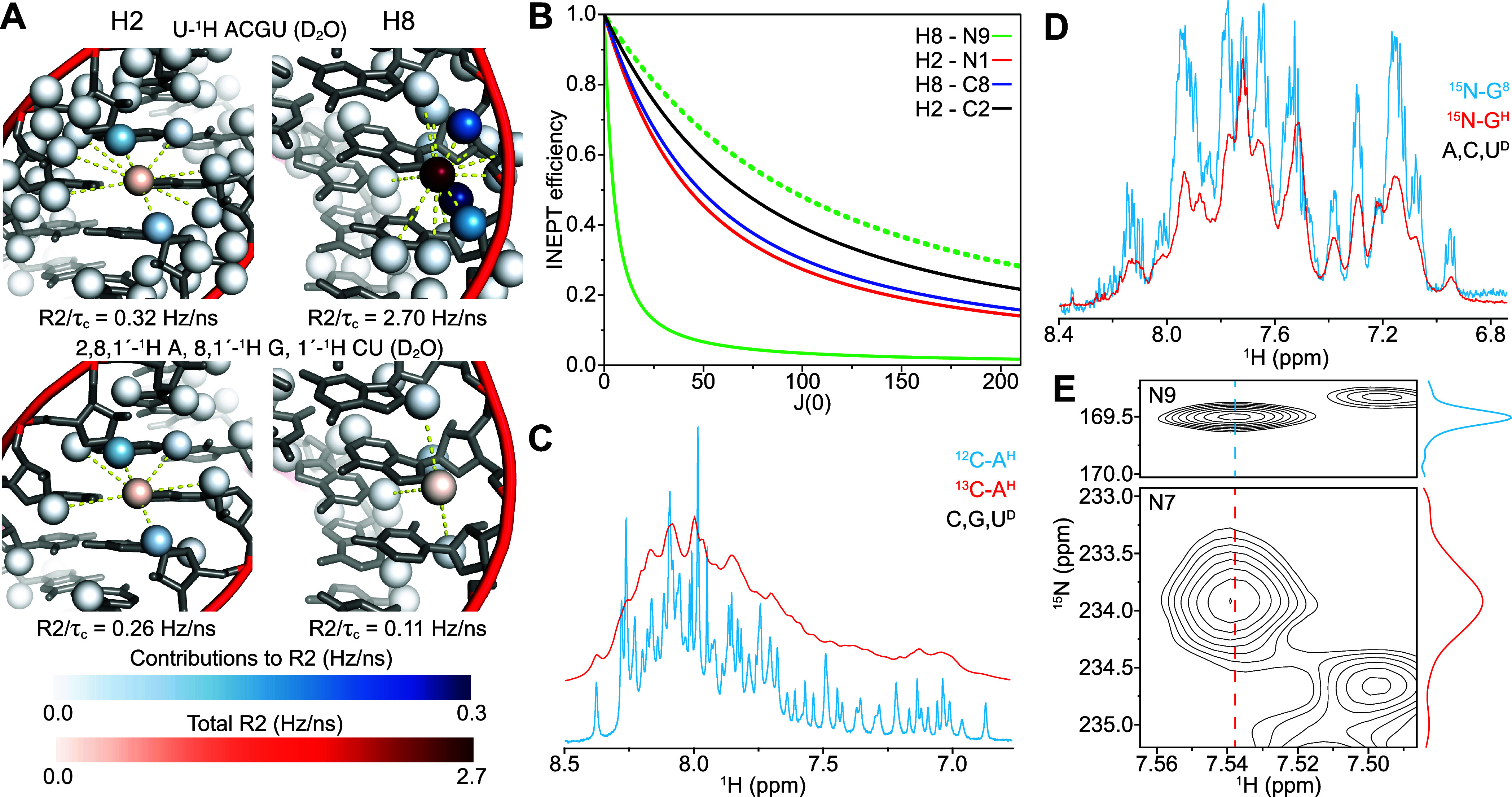
(A) Sources of dipolar relaxation in RNAs. When dissolved in D_2_O, adenosine H2 nuclei are relatively well-isolated from nearby protons even in fully protiated RNA (upper left), while purine H8 nuclei are in close proximity to ribose protons which contribute to its high transverse relaxation rate (upper right). These contributions are removed in RNAs containing heavily deuterated ribose (lower). (B) Limiting INEPT efficiencies for H8–N9 (green), H2–N1 (red), H8–C8 (blue), and H2–C2 (black) correlations with fully protiated (solid lines) or perdeuterated (dashed lines) ribose. (C) ^1^H NMR spectra of RRE232, with either [*U*-^13^C]-A, [*U*-^2^H]-CGU, (red) or [*U*-^1^H]-A, [*U*-^2^H]-CGU (blue) labeling (Supporting Information Figure 1A,B). Incorporation of ^13^C leads to severe ^1^H line broadening. (D) ^1^H NMR spectra of RRE232, with either [*U*-^15^N]-G, [*U*-^2^H]-A,C,U (red), or [*U*-^15^N; 1′,2′,3′,4′,5′,5″-^2^H]-G, [*U*-^2^H]-ACU (blue) labeling (Supporting Information Figure 1C,D). The central component of the triplet can be resolved from the satellites in the perdeuterated ribose spectrum (separation ∼8 Hz), illustrating the slow transverse relaxation with this labeling. (E) Nitrogen relaxation is dominated by chemical shift anisotropy, which is ∼3-fold smaller in purine N9 than other purine base nitrogens, leading to ∼10-fold reduced transverse relaxation rates.

We used the structure of the stem region of helix-35 of *Escherichia coli* 23S rRNA (PDB ID 2GBH)^30^ to estimate the effect of isotope composition on relaxation of aromatic protons in A-form RNA elements (Figure 1A). We find *R*2^dd^/τ_*c*_ of 2–4 Hz/ns for purine H8 and pyrimidine H6 nuclei, compared to ∼0.8 Hz/ns for adenosine H2 nuclei in fully protiated RNA molecules. This effect is emphasized when exchangeable protons are replaced with deuterium, a common strategy that is easily achieved by dissolution in D_2_O. This has little effect on H8 and H6 transverse relaxation but reduces *R*2/τ_*c*_ to ∼0.4 Hz/ns for adenosine H2 nuclei, primarily due to exchange of the nearby imino proton in A·U base pairs. Despite this improvement, increased solvent viscosity and extended proton T1 relaxation times mean that the use of H_2_O may be preferable, as described below. The major contributors to H8 and H6 transverse relaxation are intraresidue and preceding residue ribose protons, accounting for 2–3.6 Hz/ns. These protons can be replaced with deuterons using efficient enzymatic approaches for ribose–nucleobase coupling.^43−46^ Using perdeuterated ribose, their contribution to transverse relaxation rates should decrease by a factor of  such that their effect is negligible (<0.1 Hz/ns) (Figure 1A). Introduction of ^13^C has a dramatic effect on relaxation rates, with the one-bond attached carbon alone contributing ∼4.5 Hz/ns for all nonexchangeable base protons. In contrast, the effect of ^15^N incorporation is negligible, with total contributions <0.05 Hz/ns for all nonexchangeable base protons.

In the slow motion limit, the contribution of chemical shift anisotropy to transverse relaxation is given by
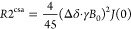


With Δδ the chemical shift anisotropy and *B*_0_ the external magnetic field. This is usually relatively small for protons in RNA but can be similar to the contribution of dipolar coupling for some of the heavily deuterated labeling schemes considered here. Using previously calculated values for Δδ,^47^ at 14.1 T (600 MHz proton Larmor frequency) we expect *R*2^csa^/τ_*c*_ of ∼0.15 Hz/ns for adenosine H2, and ∼0.05 Hz/ns for all other nonexchangeable base protons.

We analyzed the limiting INEPT transfer efficiencies with increasing *J*(0) for various heteronuclear correlations and optimal transfer times, considering only losses from proton transverse relaxation due to dipolar coupling and chemical shift anisotropy (at 600 MHz), ignoring any cross-correlations (Figure 1B). Despite fast proton relaxation upon ^13^C incorporation, the large single bond ^13^C–^1^H coupling allows relatively high transfer efficiency for this correlation, even for large RNAs. However, the sensitivity of detection decreases linearly with *R*2. To demonstrate this effect, we compared ^1^H NMR spectra for RRE232 with either [*U*-^1^H]-A, [*U*-^2^H]-CGU, or [*U*-^13^C]-A, [*U*-^2^H]-CGU labeling (Figure 1C, Supporting Information Figure 1A,B). As expected, we see significantly broadened signals with ^13^C incorporation, with outlying signals difficult to distinguish from the baseline even with ^1^H 1D experiment times of up to 50 min.

**Figure 2:**
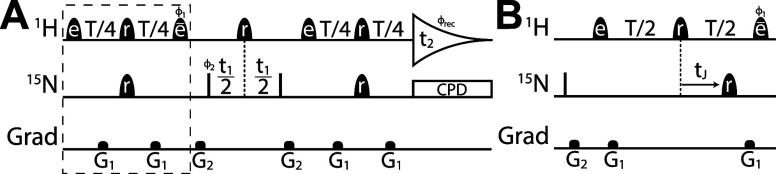
(A) Selective HSQC pulse sequence for measuring H8–N9 correlations. Narrow filled bars represent 90° pulses, while filled half-ellipsoids represent shaped pulses, with *e*, *e̅*, and *r* indicating EBURP2, time-reversed EBURP2, and ReBURP pulses, respectively.^48^^1^H shaped pulses have a duration of 2 ms (at 600 MHz ^1^H frequency) and are typically centered at 7.8 ppm. ^15^N shaped pulses have a duration of 2 ms and are centered at ∼169 ppm. All pulses have phase *x* unless otherwise indicated. Delay *T* is set at 1/*J*. Phase cycling: ϕ_1_ = *y*, −*y*; ϕ_2_ = 2(*x*), 2(−*x*); ϕ_rec_ = *x*, −*x*, *x*, −*x*. In addition, ϕ_2_ is incremented in States-TPPI manner to achieve quadrature detection in the F1 dimension.^49^*G*_1_ and *G*_2_ act as refocusing and homospoil gradients, respectively. (B) Pulse sequence element for measuring coupling which replaces the boxed element in panel (A). *t*_J_ is incremented giving a signal modulated by ^2^*J*_HN_. Full refocusing of heteronuclear coupling evolution is expected during the ReBURP pulses^50^ such that the effective dephasing time is *T* – *t*_J_.

Slow adenosine H2 relaxation makes H2–N1 and H2–N3 correlation experiments reasonably sensitive even in large, fully protiated RNAs,^42,51^ but the restriction to adenosines is a significant limitation, particularly for RDC order tensor analysis^52^ that we expect to be important in large RNAs. In this approach the average alignment of each helix is determined independently using RDCs from Watson–Crick base paired residues. Helical elements lacking sufficient A·U base pairs (such as S5 from RRE232, Figure 6A) therefore require RDCs in other residue types to be determined for this analysis. Similar two-bond ^1^H–^15^N correlation approaches applied to purine H8–N7/N9 nuclei could expand coverage to all G·C and A·U base pairs, but the combination of fast H8 relaxation and small ^2^*J*_HN_ couplings make utilizing these correlations impractical in fully protiated RNAs, even for quite small systems. A potential solution is to employ samples with deuterated ribose moieties, which can substantially reduce the H8 transverse relaxation rate (Figure 1D). Transverse relaxation of nitrogen bases in nucleic acids is dominated by the chemical shift anisotropy which for N9 (∼124 ppm) is significantly smaller than for other purine nitrogens (310–370 ppm),^53^ such that at 14.1 T (600 MHz proton Larmor frequency) we expect *R*2/τ_*c*_ of ∼0.2 Hz/ns for N9 compared to 1.2–1.8 Hz/ns for other purine nitrogens. To test this prediction, we incorporated ^15^N-labeled NTPs with perdeuterated ribose into RRE232 and obtained heteronuclear correlation spectra using a ^15^N-selective HSQC experiment (Figure 2A). Comparison of N9 with N7 and adenosine N1 and N3 nuclei revealed significantly narrower N9 line widths, in accordance with predictions (Figure 1E, Supporting Information Figure 2). Subsequent studies therefore focused on H8–N9 correlation spectra, which may also report on structure and conformational dynamics due to the expected sensitivity of the N9 chemical shift to both the torsion angle about the glycosidic bond^55^ and sugar pucker,^56^ with those in the *syn* conformation shifted upfield relative to the *anti* conformation,^55^ and with large upfield shifts for C2′-endo (S-type) relative to C3′-endo (N-type) ribose ring puckers.^56^

**Figure 3:**
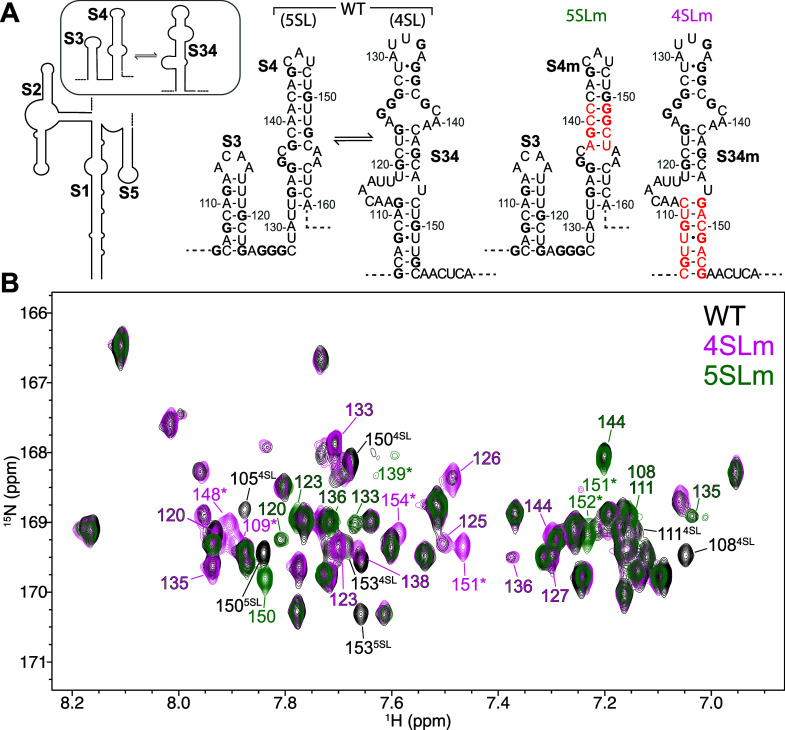
(A) RRE232^WT^ exists as two conformers in solution. We introduced mutations which have been proposed to stabilize each conformer.^54^ (B) H8–N9 correlation spectra of RRE232^WT^ (black), RRE232^4SLm^ (pink), and RRE232^5SLm^ (green) are remarkably consistent, with spectra of each of the mutants showing a subset of signals of RRE232^WT^. Residues incorporated by stabilizing mutations are denoted by an asterisk. Residues removed by stabilizing mutations are denoted by their conformer in superscript.

We anticipate that H8–N9 correlation spectra will be broadly useful as a fingerprint for large RNAs, similar to the amide ^1^H–^15^N HSQC in proteins. Assessing sample quality and the impact of conditions and mutations on RNA conformation should be simplified greatly using these approaches. For example, in-gel SHAPE probing has identified two major conformations for RRE232, comprising either 4 or 5 stems surrounding a central bulge,^54^ but other structural analyses have been interpreted in terms of a single secondary structure.^57^ To confirm the presence of these conformers in solution, we made [*U*-^15^N; 1′,2′,3′,4′,5′,5″-^2^H]-G, [*U*-^2^H]-ACU labeled samples (Supporting Information Figure 1C) of RRE232 with no mutations (RRE232^WT^) or containing mutations designed to stabilize the 5 stem (RRE232^5SLm^) or 4 stem (RRE232^4SLm^) conformations in turn (Figure 3A). We measured H8–N9 correlation spectra and used DEEP picker^58^ with default options to identify 61/67 and 61/66 guanosine signals in the H8–N9 correlation spectra for RRE232^5SLm^ and RRE232^4SLm^ respectively. In the RRE232^WT^ spectrum 72 signals were identified from 66 guanosines, which we interpreted as evidence for more than one conformer present in solution. By comparing the H8–N9 correlation spectra (Figure 3B) it was readily apparent that both RRE232^5SLm^ and RRE232^4SLm^ recapitulate the overall fold of RRE232^WT^, with identical chemical shifts for the majority of signals, only differing for signals from the regions expected to form different secondary structures. The RRE232^WT^ spectrum contains signals from each of RRE232^5SLm^ and RRE232^4SLm^ at approximately half the intensity of the mutant spectra for these regions, suggesting an approximately even mixture of the two conformers.

**Figure 4:**
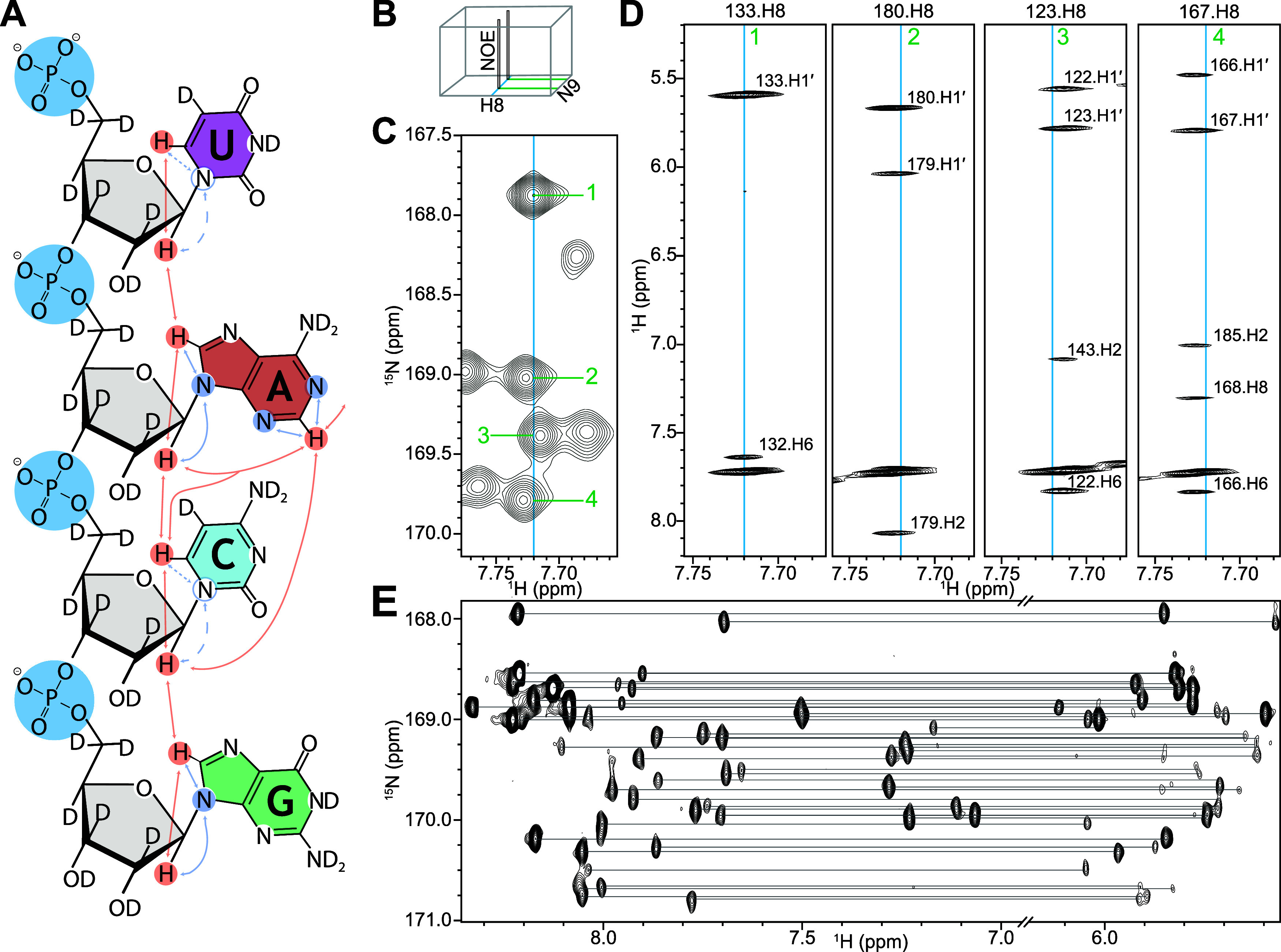
(A) Heteronuclear *J*-coupling (blue) and ^1^H–^1^H NOE (red) correlations available with this labeling scheme. Possible extension to pyrimidines shown with dashed lines. (B) Selective 3D ^15^N-edited NOESY allows unambiguous N9 assignment. (C) In the H8–N9 HSQC, it is not possible to discriminate between multiple signals at 7.72 ppm. (D) By incorporating H1′, NOEs that allow assignment of each nitrogen can be measured. (E) Two-bond H1′–N9 correlations unambiguously identify intraresidue connectivity.

Chemical shift assignments for H8 determined using existing selective deuteration approaches^59−61^ are sufficient for unambiguous assignment of outlying signals in the heteronuclear correlation spectra. For overlapping H8 signals, a method is required to discriminate between them and assign chemical shifts for their attached nitrogen. The proximity of H8 to intraresidue and preceding H1′ nuclei means that we expect to be able to detect H8–H1′ NOEs without a large detrimental effect on H8 transverse relaxation (<0.1 Hz/ns). We therefore prepared [*U*-^15^N; 2′,3′,4′,5′,5″-^2^H]-AG, [5,2′,3′,4′,5′,5″-^2^H]-CU RRE232^4SLm^ (Figure 4A, Supporting Information Figure 1E) and recorded ^1^H–^1^H NOESY (Figure 4B–D) and H8/H1′–N9 correlation spectra (Figure 4E). Because of the relatively small two-bond H1′–N9 coupling (∼3 Hz^41^) not all signals were present in the H8/H1′–N9 correlation spectrum designed for concurrent H8–N9 and H1′–N9 magnetization transfer (Supporting Information Figure 3). We therefore mainly relied on existing ^1^H chemical shift assignments in combination with a 3D ^15^N-edited NOESY-HSQC (Figure 4B) for unambiguous ^15^N chemical shift assignment of overlapping signals (Figure 4C,D), and used the H8/H1′–N9 signals for additional confirmation. In the absence of existing ^1^H chemical shift assignments unambiguous intraresidue H8–H1′ correlations may be especially valuable. In these cases experiments with separately optimized H1′–N9 and H8–N9 INEPT transfers may be preferable. Additional NOEs to adenosine H2 and H8 and pyrimidine H6 nuclei (Figure 4D) aided assignment, and suggests to us that the additional incorporation of ^15^N-labeled pyrimidines may allow chemical shift assignment via an uninterrupted NOESY walk in a single sample via H6–N1 or H5–N1 and H1′–N1 couplings (Figure 4A), despite their small predicted magnitude (∼2–5 Hz).^47^

**Figure 5:**
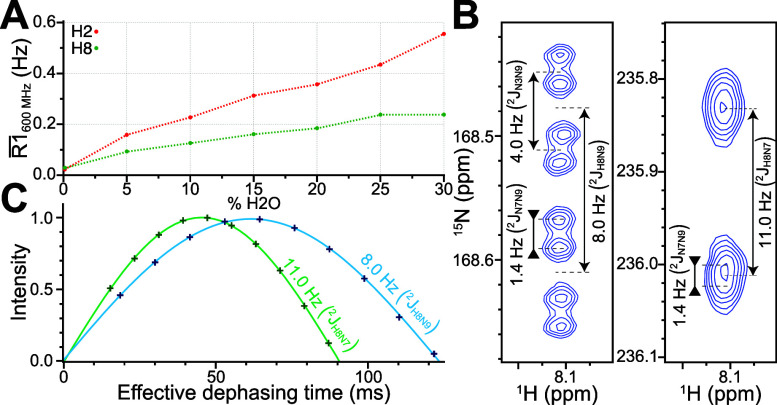
(A) R1 increases approximately linearly with proportion of H_2_O, such that longitudinal relaxation times decrease from 35–45 s in ∼100% D_2_O to 2–4 s in 30% H_2_O. (B) Selective quantitative J experiments (Figure 2B) were performed on [*U*-^15^N; 1′,2′,3′,4′,5′,5″-^2^H]-GTP and ^2^*J*_H8N7_ and ^2^*J*_H8N9_ were extracted by nonlinear least-squares fit giving 11.0 and 8.0 Hz, respectively. This experiment was repeated with a range of selective pulse offsets as well as deliberate pulse miscalibrations with negligible effect on the extracted coupling (Supporting Information Figure 5). (C) Couplings measured from high resolution selective HSQC without decoupling during t1 evolution give ^2^*J*_H8N7_ and ^2^*J*_H8N9_ of 11.0 and 8.0 Hz respectively, confirming the accuracy of the quantitative *J* experiment.

Although sparse protonation is effective in reducing proton transverse relaxation rates it can also decrease longitudinal relaxation rates, decreasing sensitivity per unit time due to requisite long relaxation delays between transients. Longitudinal spin relaxation enhancement techniques (L-optimization) which exploit efficient energy transfer to nearby unexcited protons can substantially decrease T1 relaxation times.^62−64^ It has previously been noted that recording experiments in H_2_O while incorporating band-selective pulses to avoid perturbing water polarization can enhance sensitivity in ^1^H–^13^C experiments in RNAs,^28^ presumably due to nearby exchangeable protons. Sequential and intraresidue 2′-hydroxyl protons are relatively close to purine H8 protons (4–5 Å), and due to their fast solvent exchange their spin polarization should be recovered quickly when employing pulse sequences that avoid polarization of water. To test this we measured the average ^1^H R1 as a function of H_2_O/D_2_O ratio while employing band selective ^1^H pulses throughout, and selecting for either H8 or H2 signals via their correlation to N9 and N3 respectively (Figure 5A). After repeated rounds of lyophilization and dissolution in 99.96% D_2_O with a [*U*-^15^N; 1′,2′,3′,4′,5′,5″-^2^H]-A, [*U*-^2^H]-CGU labeled RRE232 sample (Supporting Information Figure 1F), we measured T1 relaxation times of ∼44 s for H2 and ∼34 s for H8 nuclei (Figure 5A, Supporting Information Figure 4). As little as 5% H_2_O led to a significant reduction in the measured T1 (H2: ∼6 s, H8: ∼11 s), with little effect on spectral quality, with further improvement on subsequent addition to ∼2 s and ∼4 s for H2 and H8 respectively with 30% H_2_O. The decreased viscosity in H_2_O compared to D_2_O should also lead to decreased correlation times and thereby reduced transverse relaxation.^28^ For H2 correlation spectra, the proximity of uridine H3 nuclei in Watson–Crick base pairs may counter this improvement, contributing ∼0.5 Hz/ns to H2 relaxation, but we expect the impact of exchangeable protons on H8 T2 relaxation to be minimal (∼0.1 Hz/ns).

Two-bond H8–N correlations can also be exploited for determining RDCs, measured as the differences in couplings observed during isotropic rotational tumbling and upon partial molecular alignment (here, *J* refers to total measured coupling, unless otherwise stated). Couplings were measured using a modified quantitative *J*-like experiment^65^ (Figure 2B), in which the total effective coupling evolution time is varied by adjusting the placement of a ^15^N ReBURP pulse during the initial INEPT delay. This pulse was offset from the ^1^H ReBURP pulse, and we assumed that all coupling evolution during these pulses was refocused, as previously described.^50^ The total effective coupling evolution time was therefore taken to be the total INEPT delay excluding both ReBURP pulses. We tested this using [*U*-^15^N; 1′,2′,3′,4′,5′,5″-^2^H]-GTP, for which ^2^*J*_H8N7_ and ^2^*J*_H8N9_ can be readily measured using a high-resolution selective HSQC without decoupling during ^15^N evolution (Figure 5B, ^15^N total acquisition time ∼1 s). We measured ^2^*J*_H8N7_ = 11.0 Hz and ^2^*J*_H8N9_ = 8.0 Hz, in agreement with previously reported values.^41^ A nonlinear least-squares fit of extracted intensities from the selective quantitative *J* experiment gave equivalent couplings, supporting the assumption that no net coupling evolution occurs during the selective pulses (Figure 5C). A parameter for any timing offset was allowed to vary during the fit and was negligible for both couplings (<0.2% of the total coupling evolution time). To test the potential impact of chemical shift offset and miscalibration of the selective pulses, a series of experiments were conducted that (i) adjusted the offset of the ^1^H and ^15^N selective pulses so that the GTP signals were near the edge of the ReBURP excitation window (600 Hz offset with a 3 ms ReBURP pulse), (ii) adjusted the ReBURP pulse lengths between 2 and 5 ms, and (iii) deliberately miscalibrated the eBurp and ReBURP pulse powers by up to 20%. In all cases the extracted couplings were within 0.1 Hz of the original values with fitted timing offsets <0.2% of the total coupling evolution time (Supporting Information Figure 5). For obtaining couplings in large RNAs we recorded spectra with total effective coupling evolution times of *T*/2 (the reference experiment) and *T* (the attenuated experiment), where *T* ≈ 1/*J*. This scheme for recording couplings during the initial INEPT transfer was introduced as part of the ARTSY approach,^28,66^ where expressions for the couplings and their expected uncertainties are related to the intensity ratio in the two experiments, *Q*
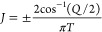
with uncertainty
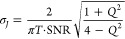


**Figure 6:**
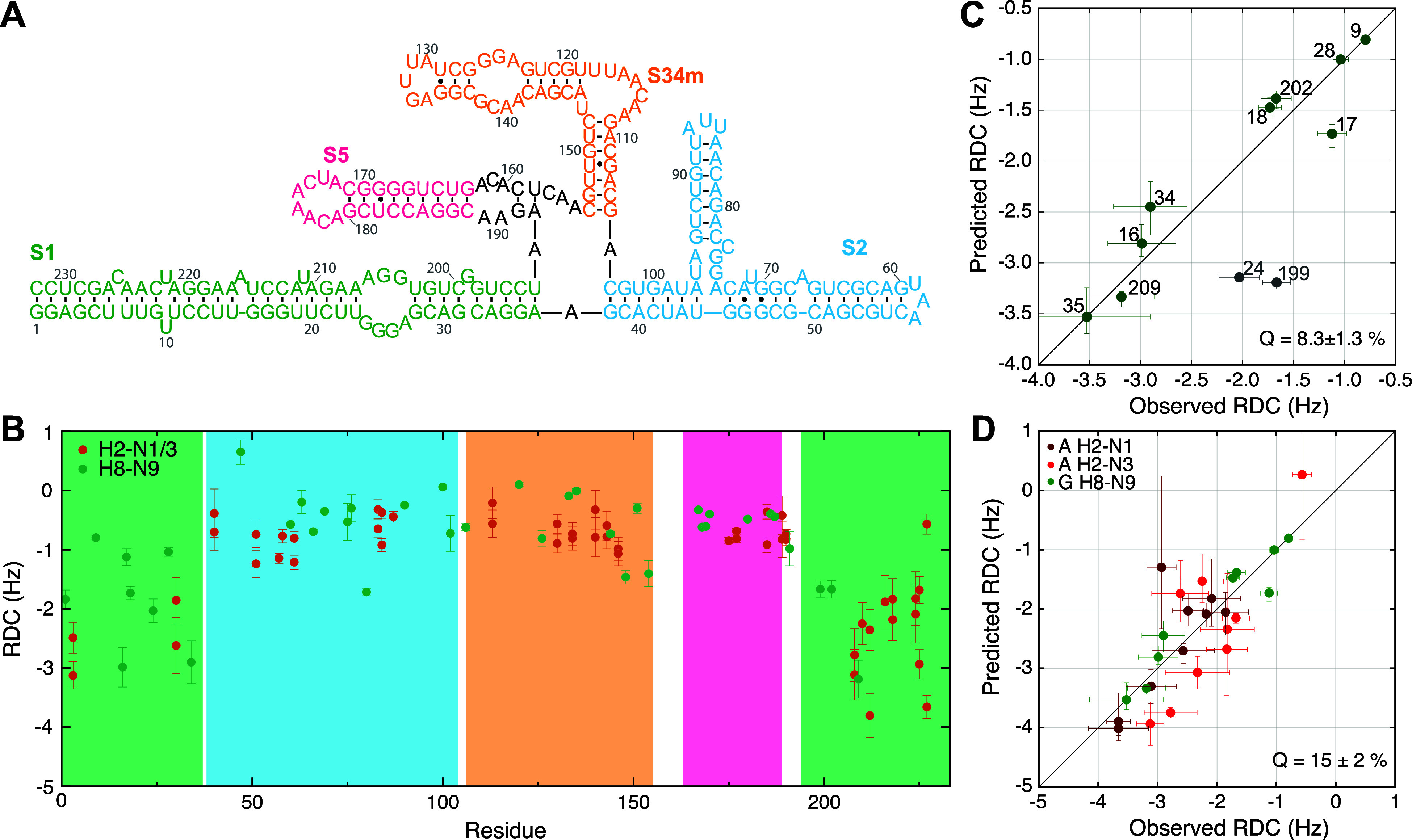
(A) NMR-derived secondary structure of RRE232^4SLm^. (B) RRE232^4SLm^ RDCs measured as described here (H8–N9, green) or using VF-HMQC (H2–N1/N3, red).^42^ RDCs are scaled to account for differences in alignment medium concentration. The estimated degree of ordering based on the size of the largest RDC is ∼3-fold higher for the long S1 A-form helix, suggesting averaging of the other elements due to interdomain motion.^68,69^ (C) Observed ^2^D_H8N9_ values for stem 1 of RRE232^4SLm^ are well correlated with back-calculated RDCs from an A-form ensemble calculated without RDC restraints (*Q* ≈ 8%). Error bars for predicted RDCs represent the maximum and minimum RDCs calculated for the ensemble. RDCs shown in gray are from residues that do not form Watson–Crick base pairs and are excluded from the fit and the calculation of *Q*-values. (D) Observed adenosine ^2^D_H2N1_ and ^2^D_H2N3_ values for stem 1 of RRE232^4SLm^ are well-correlated with back-calculated RDCs using alignment tensors derived from guanosine H8–N9 RDCs (*Q* ≈ 15%).

Where SNR is the signal-to-noise ratio of the reference experiment. The RDC is measured as the difference in couplings obtained under isotropic conditions and in the presence of an aligning medium, the total uncertainty will therefore be the root-sum-square of the uncertainties in the two experiments. If this uncertainty is more than an order of magnitude smaller than the range of the measured dipolar couplings, the uncertainty in the coordinates of the reference structure will generally dominate the residual when fitting the RDCs.^67^ When measuring isotropic couplings which fall into a small range around 8 Hz for H8–N9 correlations, an SNR of ∼25 in the reference spectrum will give noise-limited uncertainties in the derived coupling of ∼0.1 Hz when *T* = 1/*J*. Some care is required when selecting appropriate values for *T* where there is a range of couplings to be determined, as upon alignment. The SNR of the reference experiment will decrease due to incomplete INEPT magnetization transfer, as will d*Q*/d*J*. σ_*J*_ is symmetric about points where sin(π*JT*/2) = 0, so choosing *T* = 1/*J*_ave_, where *J*_ave_ represents the average of the expected couplings is natural. We analyzed the impact of the choice of *T* on the noise-limited uncertainty while accounting for the transfer efficiency for both INEPT transfers. Choosing *J*_ave_ = 12.5 Hz and assuming an SNR of 25 for signals with that coupling (and a lower SNR for other couplings) should give uncertainties less than an order of magnitude smaller than the range of the couplings for RDCs with up to a 12 Hz range (Supporting Information Figure 6), which already represents a relatively a high degree of alignment (∼0.25%).

We used this approach to measure ^2^*J*_H8N9_ for [*U*-^15^N; 1′,2′,3′,4′,5′,5″-^2^H]-G, [*U*-^2^H]-ACU labeled RRE232^4SLm^ in isotropic conditions and after alignment with ∼10.5 mg/mL Pf1 phage coat protein (Pf1).^70^ From 66 guanosines we measured 34 H8–N9 RDCs of between −3.5 and 0.7 Hz, with uncertainties of <0.5 Hz (Figure 6B), with the remainder either not measured due to overlap, incomplete chemical shift assignment, or excluded for high uncertainties. RRE232^4SLm^ was soluble to at least 1 mM with no signs of sample aggregation under isotropic conditions, but became turbid at concentrations above ∼120 μM upon addition of Pf1. While this resolved upon dilution of the RNA, the sensitivity of the aligned experiment was therefore significantly decreased, contributing to high uncertainties for some residues. Nevertheless, the additional guanosine H8–N9 RDCs measured using this approach are highly complementary to adenosine H2–N RDCs measured using existing approaches,^42^ allowing analysis of structure and dynamics for regions of the RRE that would otherwise be impossible due to the low density of adenosines in some elements (Figure 6B).

When the long helical axis is aligned, on average, in parallel with the magnetic field (as upon the addition of Pf1), H8–N9 RDCs are expected to have negative values, with a range that scales with the degree of ordering.^68,69^ RRE232^4SLm^ comprises a central bulge surrounded by 4 stems (Figure 6A). The first stem is considerably longer than the remaining stems and exhibits RDCs ∼3-fold larger, consistent with the remaining stems experiencing a lower degree of alignment due to interhelical motions, but with stem 1 relatively well ordered. We therefore used this stem for evaluation of the quality of the restraints obtained for RRE232^4SLm^. We generated an ensemble of 20 model S1 structures with an A-form geometry without incorporating any RDC restraints and fitted our measured S1 RDCs values against each member. The measured RDC values from two unpaired residues were found to be smaller than their predicted values, possibly due to internal disorder or inaccuracies in their coordinates in the model helix, while the 9 RDC values from base-paired guanosines fit well with each member of the ensemble (*Q*-values of 6–11%, Figure 6C). We next compared 9 H2–N1 and 9 H2–N3 RDCs for base-paired adenosines measured for RRE232^4SLm^ using the VF-HMQC approach^42^ with those predicted using the alignment tensors derived from fitting of the G H8–N9 RDCs, and found good agreement (*Q* values of 12–19%, Figure 6D). The guanosine H8–N9 RDC data are thus consistent with an A-form S1 helix and with RDCs measured using the VF-HMQC approach, and demonstrate the feasibility of using these experiments for measuring RDCs in large RNAs.

We anticipate that this approach will allow other NMR methods that are routinely applied to proteins and smaller nucleic acids to be applied to large RNAs, including characterization of dynamics and conformational exchange through ^15^N relaxation dispersion^71^ and CEST^72,73^ experiments, and ligand binding through measurement of chemical shift perturbation upon binding.^74^

## Conclusion

We have presented a new approach for extending heteronuclear correlated NMR experiments to large RNAs (up to at least 78 kDa). The approach enables measurement of RDC structural information over a much wider coverage of large RNAs than was previously possible using only adenosine H2 correlations. Sample quality, conformational heterogeneity, and the impact of conditions and mutations can be assessed from 2D H8–N9 correlated spectra, which can serve as a readily obtained “fingerprint” for large RNAs similar to the amide ^1^H–^15^N HSQC ubiquitously employed for proteins.

## Experimental Methods

### In Vitro Transcription

RNA molecules were produced by in vitro transcription using T7 RNA polymerase^75^ in 7.5 mL reactions, containing 50 μg of PCR-amplified DNA template, 2 mM spermidine, 80 mM Tris·HCl (pH 8.5), 2 mM DTT, 20% (v/v) DMSO, 0.5 mg T7 RNA polymerase, 10–20 mM MgCl_2_, and 3–6 mM NTPs. DNA templates are 2′-*O*-methyl-modified at the last two nucleotides of the 5′ end to improve 3′ end homogeneity of transcribed RNA.^76,77^ Labeled NTPs were purchased from Cambridge Isotope Laboratories. Reactions were incubated at 37 °C for 4–6 h before quenching by addition of EDTA. RNA was purified by electrophoresis on urea-containing polyacrylamide denaturing gels (SequaGel, National Diagnostics) using CBS scientific vertical gel system at 20 W overnight, before electroelution using the Elutrap system (Whatman) at 120 V overnight. The eluted RNAs were washed with 2 M NaCl and then desalted using a 30 kDa MWCO Amicon Ultra-4 centrifugal filter device (Millipore). The concentration of each sample was determined by measuring the optical absorbance at 260 nm.

### NMR Sample Preparation

NMR samples were prepared with 20 mM Tris–d11 buffer (pH 7.4), 140 mM KCl, and 1 mM MgCl_2_ with Roche Protector RNase inhibitor added to 1 U/μL. In the absence of Pf1 180 μL of ∼1 mM RNA was placed into 3 mm diameter NMR tubes to minimize the impact of relatively high salt concentration on sensitivity.^78^ Pf1 was then added to a final concentration of ∼10 mg/mL while diluting the RNA to ∼120 μM in 300 μL in Shigemi tubes to minimize signal losses due to low sample solubility. Pf1 concentration was estimated by the quadrupolar splitting of the D_2_O signal.^70^ Adenosine H2–N1/N3 RDCs were scaled by a factor of 9.5/9 to account for minor variation in Pf1 concentration.

### NMR Experiments

All experiments were performed at 308 K on a Bruker AVANCE III HD spectrometer at 600 MHz using a proton optimized cryogenic probe. Spectra were processed using NMRFx^79^ and NMRPipe.^80^ Chemical shifts were assigned using NMRFx, utilizing predicted chemical shifts^81,82^ and in-house plugins.

### Model Generation

S1 helical models were calculated with CYANA^83^ using a combination of torsion angle, hydrogen bond and base planarity restraints.

### Data Analysis

*J* couplings for [*U*-^15^N; 1′,2′,3′,4′,5′,5″-^2^H]-GTP were extracted using the nonlinear least-squares Marquardt–Levenberg algorithm as implemented in gnuplot (http://www.gnuplot.info). RDC fitting and back-calculation was performed using PALES.^84^

## Supporting Information Available

Nucleotide labeling schemes, comparison of H8–N9 and H8–N7 correlation spectra, comparison of H8–N9 and H8/H1′–N9 correlation spectra, longitudinal relaxation data, ^2^*J*_H8N9_ extracted under varying offsets and pulse calibrations, analysis of uncertainties for extracted couplings (Supplementary Figures 1–6) (PDF)
