# Supplementary material for: Relaxation Optimized Heteronuclear Experiments for Extending the Size Limit of RNA Nuclear Magnetic Resonance
Source: J Am Chem Soc. 2025 Mar 18;147(13):11179–88. doi: 10.1021/jacs.4c17823 (PMC11969551; doi:10.1021/jacs.4c17823)
Supplement: Supplementary file 1 — ja4c17823_si_001.pdf [file ja4c17823_si_001.pdf]

Relaxation Optimized Heteronuclear Experiments for Extending the Size Limit of RNA Nuclear Magnetic Resonance

Aarsh Shah,[†] Heer Patel,[†] Arjun Kanjarpane,[†] Michael F. Summers,[†,‡] and Jan Marchant[†,*]

[†]Department of Chemistry and Biochemistry and [‡]Howard Hughes Medical Institute, University of Maryland Baltimore County (UMBC), Baltimore, MD 21250

[*] jmarchant@umbc.edu

Supporting Information

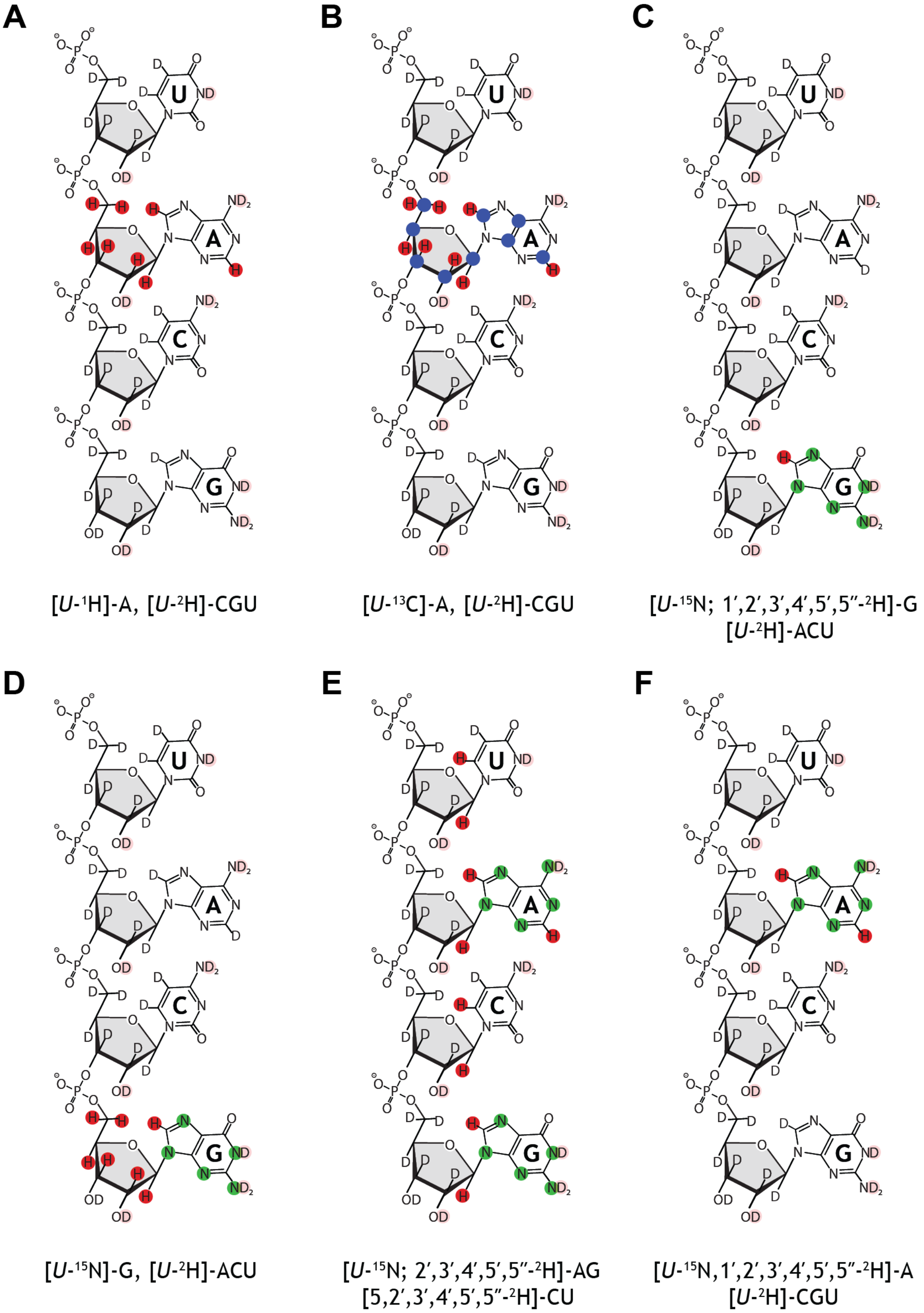

Supporting Information Figure 1. Labeled NTP combinations used in the work, as referenced throughout the text. Red circles highlight ^{1}H nuclei, pink circles exchangeable ^{1}H nuclei, blue circles ^{13}C nuclei and green circles ^{15}N nuclei.

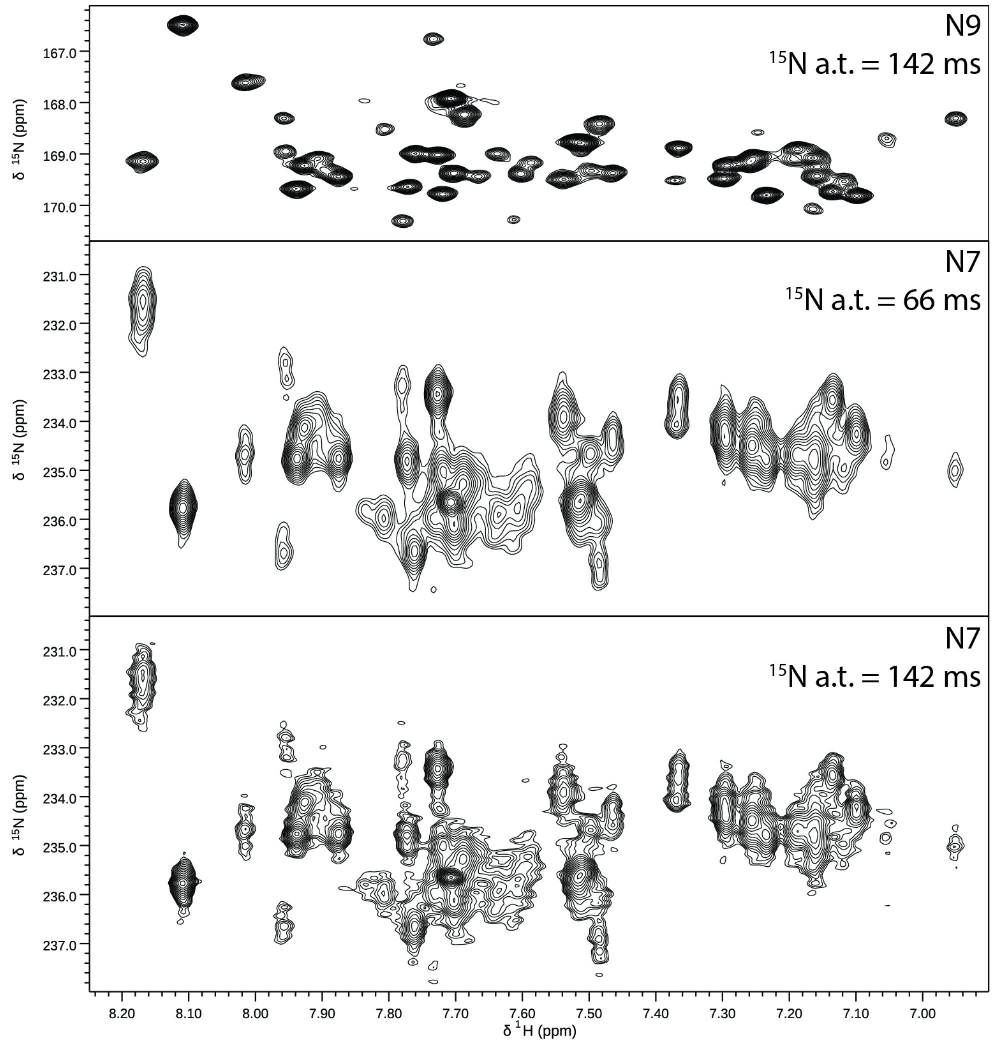

Supporting Information Figure 2. Comparison of selective H8-N9 (upper) and H8-N7 (middle, lower) correlation spectra of [*U*-^{15}N]-G, [*U*-^{2}H]-ACU RRE232^{4SLm}. The upper and lower panels show spectra acquired with identical ^{15}N acquisition time (142 ms) and apodization parameters (squared cosine bell) for direct comparison. The middle panel uses a shorter ^{15}N acquisition time (66 ms) more suitable for the broad N7 signals.

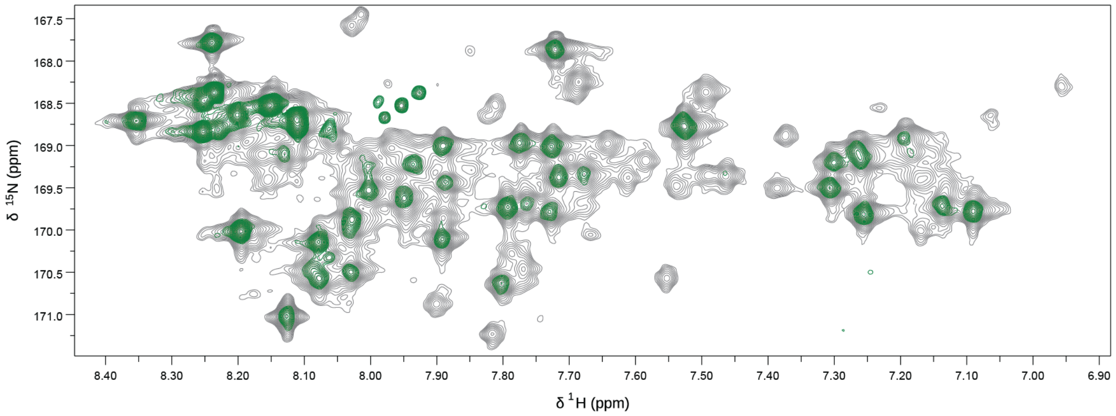

Supporting Information Figure 3. Comparison of H8-N9 (gray) and H8/H1′-N9 (green) correlation spectra for [*U*-^{15}N; 2′,3′,4′,5′,5″-^{2}H]-AG, [5,2′,3′,4′,5′,5″-^{2}H]-CU RRE232^{4SLm}. The INEPT delay is set at 50 ms for the H8-N9 and 100 ms for the H8/H1′-N9 experiment due to the anticipated ~ 3 Hz H1′-N9 coupling, such that some signals are not present in the H8/H1′-N9 spectrum.

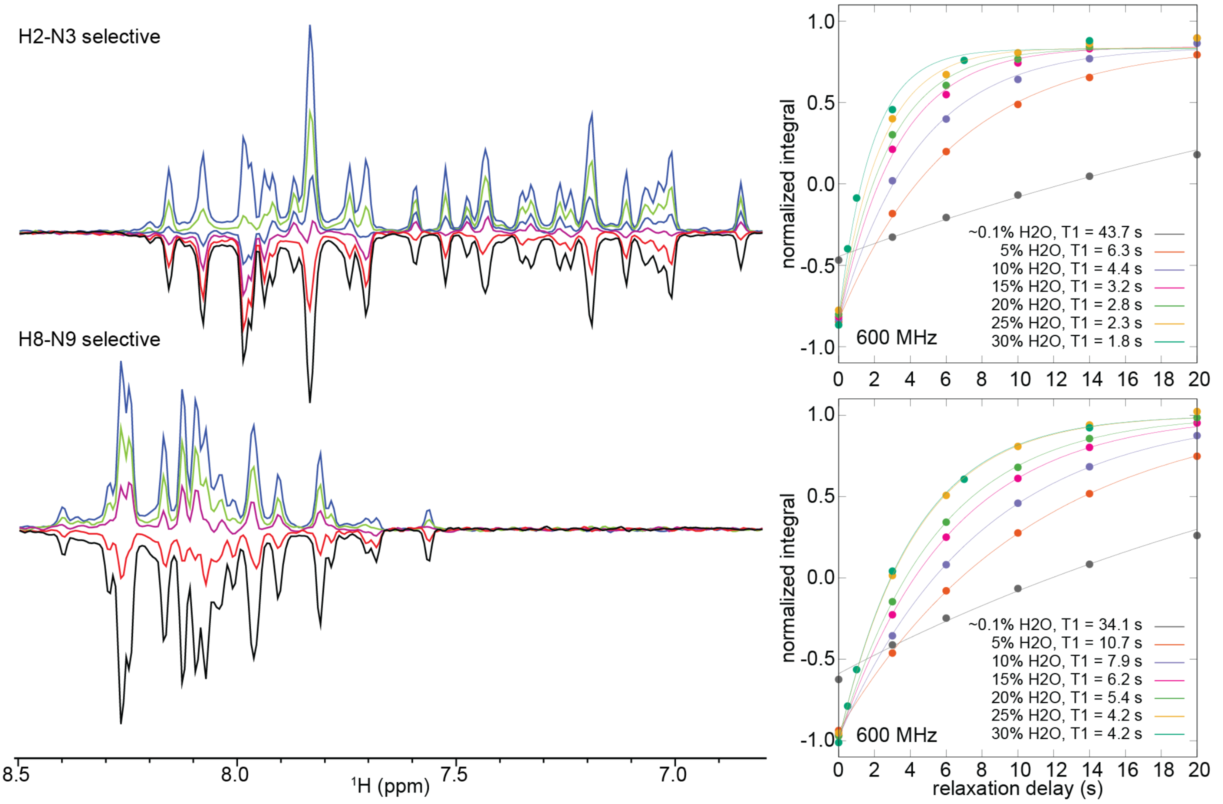

Supporting Information Figure 4. Average longitudinal relaxation times for [U-^{15}N; 1′,2′,3′,4′,5′,5″-^{2}H]-A, [U-^{2}H]-CGU labeled RRE232 with varying H_2O:D_2O ratio. Relaxation times $T1$ were determined by fitting the function $S(t) = A\left(1 - 2e^{-t/T1} + e^{-T_R/T1}\right)$ where A is a constant, t is the inversion recovery delay, and T_R is the total time per transient, included here to account for incomplete recovery in the absence of H_2O.

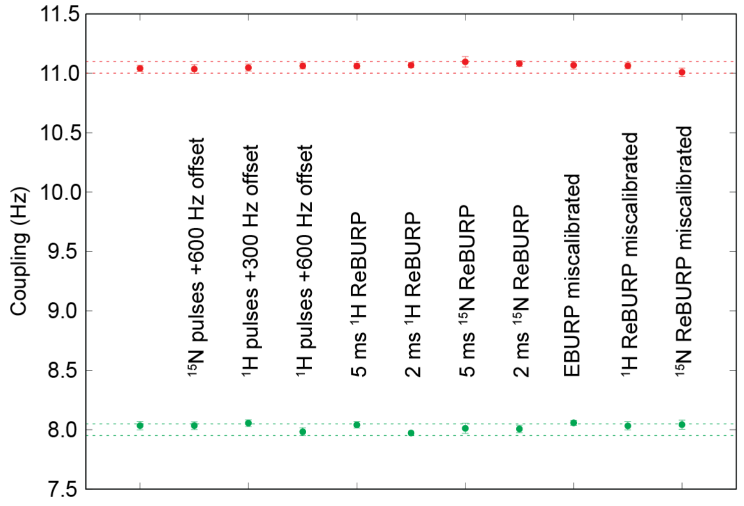

Supporting Information Figure 5. The selective quantitative-J approach is robust with varying offsets and pulse calibrations for extracting $^2J_{H8N9}$ (green) and $^2J_{H8N7}$ (red) from [U-^{15}N; 1′,2′,3′,4′,5′,5″-^{2}H]-GTP. The dashed lines show a 0.1 Hz range about the coupling determined from coupled HSQC spectra.

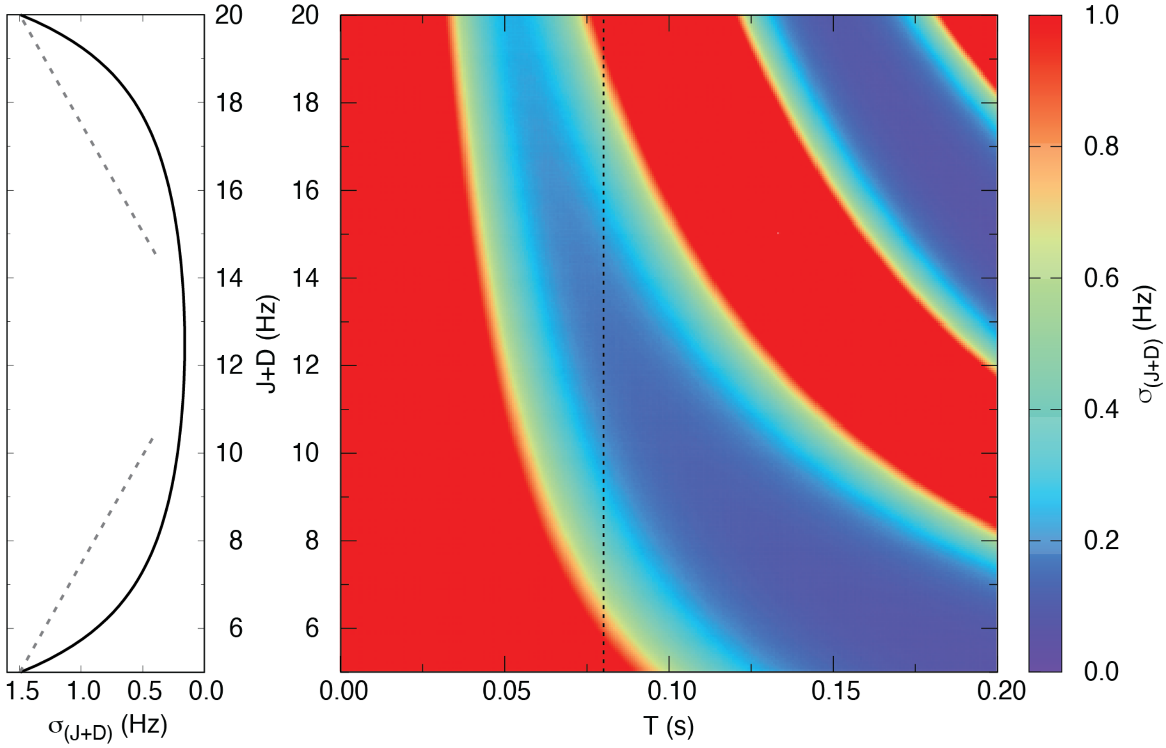

Supporting Information Figure 6. Uncertainties for extracted couplings from quantitative-J ratios according to the INEPT delay T. When calculating uncertainties it is assumed that the SNR for $J = 1/T$ is 25, and for any other coupling the SNR is reduced due to two suboptimal INEPT transfers. Relaxation losses are taken to be independent of the J coupling. The extracted slice for $T = 1/12.5$ shows that uncertainties are symmetric about $1/T$. The dashed lines indicate the limit at which the total uncertainty would be larger than the range of couplings extracted, assuming an uncertainty for the isotropic coupling of 0.1 Hz.
